# Maternal and perinatal outcome after previous caesarean section in rural Rwanda

**DOI:** 10.1186/s12884-017-1467-5

**Published:** 2017-08-25

**Authors:** Richard Kalisa, Stephen Rulisa, Jos van Roosmalen, Thomas van den Akker

**Affiliations:** 1 Department of Obstetrics and Gynecology, Ruhengeri Hospital, Musanze, Rwanda; 2 Athena Institute, VU University, Amsterdam, The Netherlands; 3 Department of Obstetrics and Gynecology, University of Rwanda, Kigali, Rwanda; 4 Department of Obstetrics, Leiden University Medical Center, Leiden, The Netherlands

**Keywords:** Elective repeat caesarean delivery, Maternal morbidity, Sub-Saharan Africa, Trial of labor, Vaginal birth after caesarean section

## Abstract

**Background:**

Offering a trial of labor (ToL) after previous caesarean section (CS) is an important strategy to reduce short- and long-term morbidity associated with repeated CS. We compared maternal and perinatal outcomes between ToL and elective repeat caesarean section (ERCS) at a district hospital in rural Rwanda.

**Methods:**

Audit of women’s records with one prior CS who delivered at Ruhengeri district hospital in Rwanda between June 2013 and December 2014.

**Results:**

Out of 4131 women who came for delivery, 435 (11%) had scarred uteri. ToL, which often started at home or at health centers without appropriate counseling, occurred in 297/435 women (68.3%), while 138 women (31.7%) delivered by ERCS. ToL was successful in 134/297 (45.1%) women. There were no maternal deaths. Twenty-eight out of all 435 women with a scarred uterus (6.4%) sustained severe acute maternal morbidity (puerperal sepsis, postpartum hemorrhage, uterine rupture), which was higher in women with ToL (*n* = 23, 7.7%) compared with women who had an ERCS (*n* = 5, 3.6%): adjusted odds ration (aOR) 1.4 (95% CI 1.2–5.4). There was no difference in neonatal admissions between women who underwent ToL (*n* = 64/297; 21.5%) and those who delivered by ERCS (*n* = 35/138; 25.4%: aOR 0.8; CI 0.5–1.6). The majority of admissions were due to perinatal asphyxia that occurred more often in infants whose mothers underwent ToL (*n* = 40, 13.4%) compared to those who delivered by ERCS (*n* = 15, 10.9%: aOR 1.9; CI 1.6–3.6). Perinatal mortality was similar among infants whose mothers had ToL (*n* = 8; 27/1000 ToLs) and infants whose mothers underwent ERCS (*n* = 4; 29/1000 ERCSs).

**Conclusions:**

A considerable proportion of women delivering at a rural Rwandan hospital had scarred uteri. Severe acute maternal morbidity was higher in the ToL group, perinatal mortality did not differ. ToL took place under suboptimal conditions: access for women with scarred uteri into a facility with 24-h surgery should be guaranteed to increase the safety of ToL.

## Background

Globally, high rates of caesarean section (CS) are an issue of public health concern [1]. According to the World Health Organization (WHO) in 2015, CS rates in women who had a previous CS ranged between 78.1 and 79.4% in high-income countries, 85.2 and 87.5% in middle-income countries and 63.2 and 72.1% in low-income countries [2]. Previous CS is one of the main indications for CS in sub-Saharan Africa [3, 4]. Even when the decision is made for a trial of labor (ToL), there are conflicting recommendations about how to manage both labor and delivery, for instance with regard to augmentation of labor. Doctor and patient preferences vary widely and fear of litigation is increasing, causing variations in clinical management [5, 6].

ToL after previous CS has been proposed to reduce CS rates [7, 8]. In sub-Saharan African countries, ToL rates vary between 37 and 97% [3, 8, 9]. Successful vaginal delivery in women with ToL in these countries stood at 70-80% [2, 10, 11]. Clinical criteria to offer vaginal delivery to women who had prior CS in most countries in sub-Saharan Africa include single previous CS, low transverse uterine scar, and single fetus [9, 12, 13]. However, risk of uterine rupture and other morbidity associated with ToL remains a concern for many practitioners [11, 12]. Some authors have argued that it is immoral to offer ToL to women in rural settings from low-resource countries [12], while others have suggested the exact opposite, meaning that withholding women ToL exposes them and their (future) children to unnecessary risks of morbidity and mortality [14–16].

The CS rate at Ruhengeri hospital, where the current study was carried out, is high (34.9%) and prior CS is a major contributing factor for repeat CS [17]. We sought to examine whether concerns about offering ToL to women in a rural sub-Saharan African setting are justified, and assessed the risk of maternal morbidity and perinatal mortality associated with ToL compared to ERCS in a district hospital in rural Rwanda.

## Methods

We conducted a retrospective cohort study of all women who had caesarean section (CS) in a previous pregnancy with a singleton infant in cephalic presentation at 36 weeks of gestation or higher in the pregnancy of study. Data were extracted from a large sample of pregnant women who were admitted for delivery at Ruhengeri maternity ward in Musanze district, Rwanda, between June 2013 and December 2014 [17]. The hospital acts as a provincial referral hospital for high-risk obstetric cases from health centers and district hospitals in the northern province. It conducts about 3500 deliveries annually, with perinatal and maternal mortality rates of 31 per 1000 live birth and 325 per 100,000 live births respectively [17]. Blood for transfusion was supplied by the regional blood bank located next to the hospital. A clinician capable of performing CS is permanently available. Although some people have private health insurance, most of the general population use community-based health insurance with an annual fee contribution of RWF 3000 (US$4.5), plus a 10% co-payment for each episode of illness. In case of shortages of drug supplies, patients are requested to procure missing items from private pharmacies.

**Fig. 1:**
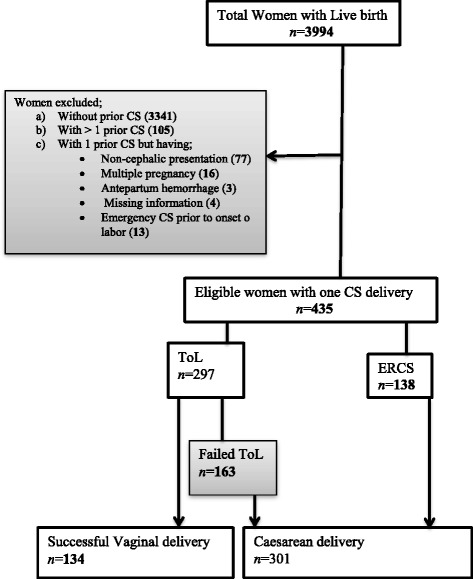
Flow chart on mode of deliveries among women underwent trial of Labor and elective repeat caesarean section

We identified potential candidates for ToL and ERCS by a process of elimination (Fig. 1). Women presenting in labor with a cervical dilatation of at least 3 cm were classified as having undergone ToL. Women with absolute contraindications to vaginal delivery in our setting (e.g. multiple pregnancy, non-cephalic presentation, intrauterine growth retardation, prior myomectomy and genital herpes) underwent ERCS. We also excluded women presenting with less than 3 cm dilatation due to the impossibility to distinguish between failed ToL and ERCS. Successful ToL was defined as vaginal delivery following ToL.

During labor, women were monitored using a partogram including regular auscultation of the fetal heart by fetoscope at least once every 30 min and regular prompting for vaginal bleeding, uterine tenderness and staining of liquor. Augmentation of labor was done by artificially rupturing the membranes, but in this specific setting oxytocic drugs were not used for fear of uterine rupture. Induction of labor was not performed. ToL was terminated if the partogram crossed the action line, if tenderness occurred at the site of the uterine scar, or in case of signs of fetal distress, the latter defined as the presence of meconium stained liquor, an irregular fetal heart beat or a heart beat of less than 120 or more than 160. Term neonates with low 5 min APGAR Score or stated as low APGAR Score but non-quantified who were encephalopathic (abnormal posture, unconscious, abnormal tone or seizures) were given a diagnosis of perinatal asphyxia. Mother and newborn were observed for at least 24 h following vaginal delivery while those women who delivered by CS and did not have complications were discharged on the fourth day after surgery.

Data were collected from medical records by two trained research assistants who were supervised by the principal investigator. For every case, information was collected regarding socio-demographic characteristics, medical history, antenatal care attendance (ANC), medical conditions diagnosed before or during current pregnancy, details of previous CS, mode of delivery, and maternal and perinatal outcome including complications.

Maternal and perinatal outcomes were compared between women who underwent ToL and ERCS. All data were entered into Microsoft Excel and transferred to STATA version 13 for analysis. Initial comparisons were done using the chi-square test for categorical data and Student’s t-test for continuous data. Maternal age, marital status, four or more ANC visits, gestational age, previous indications for CS and inter delivery interval were examined for interaction and confounding. Our analysis revealed no significant interaction among these covariates. Multivariate logistic regression analysis was used to control for simultaneous effects of covariates. Adjusted odds ratios and 95% confidence intervals were derived from the regression coefficients.

## Results

Out of 4131 women who delivered in Ruhengeri hospital, 1442 (34.9%) were via CS. Of all women who came for delivery, 435 (10.5%) had one previous caesarean section. ToL took place in 297 (68.3%) and ERCS in 138 (31.7%) women. Among women who underwent ToL, 134 (45.1%) had a successful vaginal delivery and 163 (54.9%) had an emergency CS after ToL failed (Fig. 1).

Compared to women who underwent ERCS, women with ToL were more likely to be below 30 years of age, unmarried and referred from another healthcare facility. They were also more likely to have attended more than four ANC visits, and to have had non-recurrent or unknown indications for prior CS, as well as an inter-delivery interval from the previous CS of 18 months or more. Gestational diabetes and hypertension were higher in women who delivered by ERCS (Table 1).

**Table 1:** Characteristics of women undergoing a trial of labor or elective caesarean section after previous caesarean delivery

Characteristics		ToL	ERCS	OR (95% CI)
		297 (%)	138 (%)	
Maternal Age (Years)	Less than 25	57 (19.2)	20 (14.5)	1.0
	25-30	144 (48.5)	55 (39.9)	0.9 (0.5 - 1.7)
	31-35	67 (22.6)	38 (27.5)	0.6 (0.3 - 1.2)
	Above 35	29 (9.7)	25 (18.1)	0.4 (0.2 - 0.8)
Martial status	Married	202 (68.0)	104 (75.4)	1
	Single	83 (28.0)	27 (19.6)	1.7 (1.0 – 2.8)
	Separated/widowed/divorced	12 (4.0)	7 (5.0)	0.8 (0.3 – 2.4)
Number of ANC	< 4	166 (55.9)	89 (64.5)	1
	≥ 4	131 (44.1)	49 (35.5)	1.9 (1.7 −5.2)
Educational Level	None	30 (10.1)	15 (10.9)	1
	Primary	177 (59.6)	86 (62.3)	1.1 (0.6 − 2.1)
	Secondary	50 (16.8)	20 (14.5)	1.3 (0.6 − 2.9)
	Tertiary	40 (13.5)	17 (12.3)	1.5 (0.6 − 3.8)
Health Insurance	Public (Mutuelle)	244 (82.1)	123 (89.1)	1
	Private including Others	10 (3.4)	11 (8.0)	0.4 (0.2 − 0.9)
	None	43 (14.5)	4 (2.9)	5.7 (1.9 − 16.1)
Type of referral	Self	232 (78.1)	129 (93.5)	1
	Health center/home	65 (21.9)	9 (6.5)	4 (1.9 − 8.3)
Neonatal Weights (grams)	≤ 3999	286 (96.3)	112 (81.2)	1
	≥ 4000	11 (3.7)	26 (18.8)	0.2 (0.1 − 0.3)
Maternal occupation	Housewife	101 (34.0)	47 (34.0)	1
	Subsistence Farmer	105 (35.3)	39 (28.3)	1.5 (0.9 − 2.4)
	Business	51 (17.2)	29 (21.0)	1.0 (0.5 − 1.7)
	Formal/Salaried	40 (13.5)	23 (16.7)	0.9 (0.5 − 1.7)
Parity	One	95 (31.9)	41 (29.7)	1
	Two	68 (22.9)	28 (20.3)	1.2 (0.7 − 2.2)
	Three	46 (15.5)	19 (13.8)	1.2 (0.7 − 2.4)
	Four	45 (15.2)	22 (15.9)	1.0 (0.6 − 1.9)
	Five and above	43 (14.5)	28 (20.3)	0.8 (0.5 − 1.5)
Prenatal maternal disease	None	276 (92.9)	121 (87.7)	1
	Gestational diabetes	5 (1.7)	5 (3.6)	0.5 (0.1 − 1.9)
	Hypertension	7 (2.4)	8 (5.8)	0.4 (0.1 − 1.1)
	HIV	9 (3.0)	4 (2.9)	0.8 (0.2 − 2.8)
Indication of previous CS	Recurrent	95 (32.0)	72 (52.1)	1
	Non-recurrent	123 (41.4)	27 (19.6)	3.9 (2.3 − 6.5)
	Unknown	79 (26.6)	39 (28.3)	1.7 (1.1 − 2.8)
Inter-delivery interval from prior CS (months)	≤ 18	85 (28.6)	64 (46.4)	1
	18 to 36	121 (40.8)	65 (47.1)	1.5 (1.9 − 2.3)
	≥ 36	91 (30.6)	9 (6.5)	8.3 (3.9 − 17.6)

CI denotes confidence interval. Odds ratios express the likelihood that women will choose to undergo a ToL, as compared to ERCS

Women who underwent ToL in case of a previous CS that was done for non-recurrent indications such as fetal distress, antepartum hemorrhage, multiple pregnancy, pregnancy-induced hypertension or mal- presentation had a higher vaginal birth after caesarean (VBAC)-rate (74 out of 123, 60.2%, *p* = 0.03) compared to 38 out of 95 women (40%, *p* = 0.06) whose prior indication was prolonged labor (Table 2). The main causes of failed ToL were fetal distress and prolonged labor (Table 3).

**Table 2:** Indication of prior caesarean and mode of delivery in index pregnancy among women undergoing a trial of labor

Indications	Trial of Labor		*P*-value
	Successful134 (%)	Failed 163 (%)	
Prolonged Labor	38 (28.4)	57 (35.0)	0.06
Non-recurrent	74 (55.2)	49 (30.0)	0.03
*Fetal distress*	36 (26.9)	17 (10.4)	0.013
*Malpresentation*	27 (20.1)	8 (4.9)	0.002
*Others*	11 (8.2)	24 (14.7)	0.041
Unknown	22 (16.4)	57 (35.0)	0.000

Others; successful had 2 pregnancy induced hypertension, 4 twin pregnancy and 5 Antepartum hemorrhage while in failed had 9 twin pregnancy, 8 Antepartum hemorrhage and 5 pregnancy induced hypertension

**Table 3:** Indications for failed trial of labor or elective repeat caesarean section

Indications	Failed ToL	ERCS
	163 (%)	138 (%)
Prolonged Labor	52 (31.9)	-
Fetal distress	81 (49.7)	-
Malpresentation	-	36 (26.1)
Tender uterine scar	25 (15.3)	-
Uterine rupture	5 (3.1)	-
Breech	-	41 (29.7)
Macrosomia	-	21 (15.2)
Post term	-	15 (10.9)
Intrauterine growth retardation	-	5 (3.6)
Prior myomectomy	-	12 (8.7)
Genital herpes	-	8 (5.8)

Twenty-eight out of all 435 women with a scarred uterus (6.4%) sustained maternal morbidity and were significantly higher in women with a TOL (*n* = 23, 7.7%) compared with women who had an ERCS (*n* = 5, 3.6%): (adjusted odds ratio (aOR) 1.4; CI 1.2–5.4). However, there was a non-significant trend towards severe maternal morbidities in women with ToL and ERCS: Puerperal sepsis (*n* = 10/297; 3.4%, versus 3/138; 2.2%: adjusted OR 1.9; CI 0.5–7.1), postpartum hemorrhage (*n* = 8/297; 2.7%, versus 2/138; 1.4%: adjusted OR 3.0; CI 0.6–14.5) and uterine rupture (*n* = 5/297; 1.7%, versus 0/138; 0%) respectively. Of the 13 cases of puerperal sepsis, two (*n* = 2/134; 1.5%) occurred in women who had a successful ToL and eight (*n* = 8/163; 4.9%) in women in whom ToL failed, compared to three (*n* = 3/138; 2.2%) women who had undergone ERCS. Among the ten cases of postpartum hemorrhage, six had a successful vaginal delivery among whom four developed uterine atony. All five women who had scar ruptures in ToL had been transferred to hospital; three were referred from health centers with suspected uterine rupture and two were failed home births. One woman underwent hysterectomy for uterine rupture with severe hemorrhage. The frequencies of sepsis, hysterectomy and transfusion did not differ significantly between the groups after adjusting for confounders. There were no maternal deaths (Table 4).

**Table 4:** Maternal and perinatal complications after outcome of labor

Complications	ToL 297 (%)	ERCS 138 (%)	Unadjusted OR (95% CI)	Adjusted OR (95% CI)^a^
Maternal				
Total Morbidities	23 (7.7)	5 (3.6)	2.2 (1.8 - 6.7)	1.4 (1.2 – 5.4)
Postpartum hemorrhage	8 (2.7)	2 (1.4)	1.9 (0.4 - 8.9)	3.0 (0.6 - 14.5)
Puerperal sepsis	10 (3.4)	3 (2.2)	1.6 (0.4 - 5.7)	1.9 (0.5 - 7.1)
Ruptured uterus	5 (1.6)	0 (0.0)	-	-^b^
Blood transfusion	2 (0.6)	1 (0.7)	0.9 (0.1 - 10.3)	0.7 (0.1 - 8.8)
Hysterectomy	1 (0.3)	0 (0.0)	-	-^b^
At least one maternal complication	7 (2.3)	4 (2.8)	2.2 (0.8 - 6.0)	2.4 (0.9 - 7.8)
Perinatal				
Total admissions to NICU	64 (21.5)	35 (25.4)	0.8 (0.5 - 1.3)	0.8 (0.5 - 1.6)
Birth Asphyxia	40 (13.4)	15 (10.9)	1.3 (0.7 - 2.4)	1.9 (1.6 - 3.6)
Death (Rate/1000)	8 (26.9)	4 (28.9)	0.9 (0.3 - 3.1)	0.4 (0.2 - 2.3)
Fresh stillbirth (Rate/1000)	5 (16.8)	1 (7.2)	2.3 (1.3 - 20.3)	1.4 (1.1 - 15.5)
Neonatal deaths before 24 h (Rate/1000)	2 (6.7)	3 (21.7)	0.3 (0.1 - 1.8)	0.3 (0.2 - 2.1)
Neonatal deaths after 24 h(Rate/1000)	1 (3.4)	0 (0.0)	-	-^b^

^a^Odds ratios have been adjusted for maternal age, marital status, antenatal visits, gestational age, previous CS indications and inter-delivery interval from prior CS. CI denotes confidence interval. Odds ratios express the likelihood of complications among the women who had a ToL as compared to ERCS

NICU, neonatal intensive care unit

-^b^ Denotes not applicable

There was no difference in neonatal admissions between women who underwent ToL (*n* = 64/297; 21.5%) and those who delivered by ERCS (*n* = 35/138; 25.4%: aOR 0.8; CI 0.5–1.6). The majority of admissions were due to perinatal asphyxia that occurred more often in infants whose mothers underwent ToL (*n* = 40, 13.4%) compared to those who delivered by ERCS (*n* = 15, 10.9%: aOR 1.9; CI 1.6–3.6). Perinatal mortality was similar among infants whose mothers had ToL (*n* = 8; 27/1000 ToLs) and infants whose mothers underwent ERCS (*n* = 4; 29/1000 ERCSs). The frequency of fresh stillbirths was higher among women with ToL (*n* = 8; 16.8/1000 ToLs) than among women who underwent ERCS (*n* = 4; 7.2/1000 ERCSs: aOR 1.4 CI 1.1–15.5). Among the five fresh stillbirths in the ToL group, three occurred in cases of uterine rupture (Table 4).

## Discussion

Our results add importantly to the literature about the safety of VBAC, since this is one of the few studies from low-income settings. The higher severe acute maternal morbidity and poorer perinatal outcomes in ToL, combined with the fact that many of these women start laboring far from hospital without any previous counseling or risk education, stresses the need to increase the safety of ToL in such settings.

The CS rate for this cohort was much higher than the WHO recommended CS rates of 5-15% [1, 18]. Therefore, we acknowledge the importance of offering ToL among other measures in order to prevent unnecessary CSs, which are potentially harmful and costly to mothers and health care systems [19]. The prevalence of one previous CS in our study was 10.5%, which is comparable to that of a similar setting in Tanzania [20]. This high prevalence may be due to the fact that it is policy to refer all high-risk cases.

Our rate of successful vaginal delivery was similar to findings from sub-Saharan countries [9, 21] but below the 50-80% range reported by other authors from similar settings [8, 13]. This rate could be also partly explained by the differences in referral and clinical practice gaps. Use of oxytocin for augmentation is not used in women undergoing ToL in our setting [22–24]. In general, it appears that clinicians easily opt for repeat CS [21].

Our findings show that ToL occurred most often among women who were less than 30 years of age, unmarried and who had attended more than four antenatal care (ANC) visits. This may be due to the fact that unmarried women might not have the financial means to cover the cost of caesarean section or due to the fact that they may not have the power to negotiate ERCS [25]. Additionally, prior non-recurrent CS indications tended to have more successful ToLs as compared to recurrent indications such as failure to progress [20]. Women who had unknown indications for their previous CS more often had successful ToLs, which highlights the need to evaluate such women with more precision before subjecting them to ERCS. Future unknown CS indications may be prevented by improving medical data recording.

There was significant difference in the occurrence of total maternal morbidities between women who underwent ToL compared to ERCS, although there was a non-significant trend towards severe maternal morbidities in women with ToL and ERCS. This might have been due to strict local protocol which did not allow induction with oxytocic drugs for fear of uterine rupture or women who had failed ToLs referred from other health facilities [24, 26]. Indeed, one in five of the women presented late in labor after failed ToL at health centers or home. This contributed to the occurrence of uterine scar ruptures and perinatal deaths possibly due to women avoiding another repeat caesarean section [27] or delays in seeking or reaching care [17, 28]. This highlights challenges rural women face due to lack of access to basic delivery care in low-income countries [29]. Pregnant women with uterine scars thus need to be encouraged to deliver in hospitals from the onset of labor [28]. In addition, clinicians should take responsibility to be present in the labor wards to offer appropriate monitoring of labor so that women and their babies can be assured favorable outcomes.

Therefore, preventive strategies are of utmost importance, such as educating pregnant women during ANC about success factors, risks and prospects of various modes of delivery, monitoring labor by correct use of partogram, augmentation of labor with oxytocin and prevention of unnecessary first and subsequent CSs performed in the second stage of labor by training, equipping and empowering midwives as well as medical officers and associate clinicians to perform vacuum deliveries.

We found a significant increase in the rate of perinatal asphyxia among the infants of women who underwent ToL as compared with infants born after ERCS. The latter occurred especially in those infants who were born after emergency CS due to failed ToL whose labors had started far from hospital [11, 26, 30]. However, our findings showed equal perinatal deaths in ToL or ERCS [8, 9, 13]. Uterine rupture contributed to half of fresh stillbirths in our study and others that were not related to the latter were probably associated with poor quality intrapartum care [31].

The strength of this study was that data were extracted from a database by trained health staff in a real clinical setting [17]. The main weakness was the fact that it involved only one center. However, we have no reason to believe that the situation in most labor units in the wider region would be much different from the situation described here. The possibility that bias affected the results of this study must be considered. Women who had a ToL by choice at the hospital or because labor started at home or were referred from other health facilities after failed ToL have characteristics that are different from women who underwent ERCS, and these differences might affect maternal and perinatal outcomes. We also recognize that the women who were classified as undergoing ToL some choose the latter upon arrival at the hospital especially for those women who were either referred from other health facilities or presented from home in advanced labor. This is likely to affect the results of ToL in a negative way. We acknowledge that during analysis it would have been better to separate ToL cases for those women who were or not referred from another health facility as estimates in risks of maternal and perinatal outcomes would be different. Another limitation is that our adjusted results may be affected by residual confounding, and also the study had limited power to detect differences in rare catastrophic outcomes.

We did not have the possibility to monitor women after leaving hospital and to look at long-term outcomes that may occur. There should be concern about the relatively increased maternal and perinatal complications. But overall, unless better evidence emerges to the contrary, there is no reason why women in low-income countries with one previous CS should not be offered an appropriately monitored ToL in a well-equipped hospital with 24-h availability of theatre facilities.

## Conclusions

At this moment, women may present for emergency CS following failed ToL from referring health facilities that do not have the 24-h surgery available [12, 14, 20]. Severe acute maternal morbidity was higher in the ToL group, perinatal mortality did not differ. Therefore, to increase its safety, we do stress that ToL should take place at facilities with 24-h surgery services, and under continuous monitoring by a skilled professional. Above all, we stress the importance of avoiding the first caesarean section by good clinical practice, including use of the partogram, augmentation with oxytocin and instrumental vaginal delivery. The impact of late presentation to care (e.g. after suspected rupture or obstructed labor) deserves closer investigation.

## Abbreviations

ANC: Antenatal care
CI: Confidence interval
CS: Caesarean section
ERCS: Elective repeat caesarean section
NICU: Neonatal intensive care unit
ToL: Trial of Labor
VBAC: Vaginal birth after caesarean
WHO: World Health Organization
